# Growth differentiation factor 15 is not associated with glycemic control in patients with type 2 diabetes mellitus treated with metformin: a post-hoc analysis of AIM study

**DOI:** 10.1186/s12902-022-01176-3

**Published:** 2022-10-22

**Authors:** Fei Gao, Cheng Li, Yufei Wang, Jingyi Lu, Wei Lu, Jian Zhou, Jun Yin, Xiaojing Ma

**Affiliations:** Department of Endocrinology and Metabolism, Shanghai Jiao Tong University Affiliated Sixth People’s Hospital, Shanghai Clinical Center for Diabetes, Shanghai Diabetes Institute, Shanghai Key Laboratory of Diabetes Mellitus, 600 Yishan Road, Shanghai, 200233 China

**Keywords:** Growth differentiation factor 15, Metformin, Glycemic control, Type 2 diabetes mellitus

## Abstract

**Background:**

Growth differentiation factor 15 (GDF15) was newly discovered to be a promising target of metformin. The study was aimed to investigate the relationship between GDF15 and glycemic control after metformin treatment in patients with type 2 diabetes mellitus.

**Methods:**

The study was a post-hoc analysis of AIM (the effect of Acarbose on glycemic variability in patients with type 2 diabetes mellitus using premixed Insulin compared to Metformin) study. The participants were randomly assigned to 12 weeks of metformin (MET) or acarbose (ACA) treatment combined with insulin. Serum GDF15 levels of 51 subjects from MET group and 53 subjects from ACA group were measured at baseline and after a 12-week treatment. Fasting plasma glucose (FPG), 2-h postprandial plasma glucose (2-h PG) and glycated hemoglobin A1c (HbA1c) were measured at baseline and endpoint.

**Results:**

After a 12-week treatment, serum GDF15 levels significantly increased in MET group [baseline vs. endpoint, 936.70 (741.00, 1205.40) pg/mL vs. 1265.20 (1027.90, 1634.00) pg/mL, *P* < 0.001], but not in ACA group [baseline vs. endpoint, 920.60 (701.45, 1332.55) pg/mL vs. 893.80 (663.25, 1284.05) pg/mL, *P* = 0.944]. However, there were no significant differences of glycemic control parameters (ΔFPG, Δ2-h PG and ΔHbA1c) between subgroups of MET group divided by median of ΔGDF15 (all *P* > 0.05). Spearman correlation coefficient and analysis of covariance after adjustment for baseline HbA1c levels showed that ΔGDF15 was not correlated with ΔFPG, Δ2-h PG and ΔHbA1c (all *P* > 0.05).

**Conclusion:**

Serum GDF15 levels were significantly elevated after metformin treatment in patients with type 2 diabetes mellitus. However, the increase was not an indicator of the glucose-lowering effect of metformin.

**Trial registration:**

Clinicaltrials.gov, NCT02438397. Registered 8 May 2015.

## Background

Metformin is the most commonly used oral antidiabetic drug [1–3], which can reduce hepatic glucose production and peripheral insulin resistance and thereby lower blood glucose levels [4–6]. Growth differentiation factor 15 (GDF15), also known as macrophage inhibitory cytokine 1, is a stress responsive cytokine [7–9]. Previous studies found that GDF15 was closely related to diabetes mellitus [7, 10, 11], while GDF15 was newly discovered to be a promising target of metformin [12, 13]. Coll et al. [13] revealed that metformin treatment was related to increased levels of circulation GDF15 in people without diabetes mellitus, while the change of GDF15 levels in metformin group was significantly corelated with weight loss. Our previous study also revealed that increased serum GDF15 was related to metabolic improvement by lifestyle intervention among young overweight and obese adults [14]. However, in addition to its association with weight control, the relationship between GDF15 and glycemic control during metformin therapy still remains unknown.

Therefore, in the present study, we measured the serum GDF15 concentrations from the AIM (the effect of Acarbose on glycemic variability in patients with type 2 diabetes mellitus using premixed Insulin compared to Metformin) study [15] to explore the association between metformin treatment, glycemic control and the change of serum GDF15 levels.

## Materials and methods

### Study design and participants

The present study is a post-hoc analysis of the AIM study. The AIM study was an open-labeled randomized clinical trial designed to investigate the effect of acarbose on glycemic variability in patients with type 2 diabetes mellitus using premixed insulin compared to metformin. The study protocol and main results of the trial have been published [15]. The AIM study was a prospective trail registered at www.ClinicalTrials.gov with clinical trial registration number NCT02438397.

In brief, the AIM study enrolled patients with type 2 diabetes using premixed insulin and the glycated hemoglobin A1c (HbA1c) levels were between 7 and 10% before randomization. Patients taking more than two oral antidiabetic drugs or taking one oral antidiabetic drug at the maximum therapeutic dose were excluded. The main inclusion and exclusion criteria were described in detail in the previously published article [15].

The eligible patients were randomly assigned to 12 weeks of metformin (MET group, *n* = 62) or acarbose (ACA group, *n* = 62) treatment combined with insulin according to the random encoder [15]. The initial dose of ACA was 50 mg three times a day at three meals and the dose was raised to 100 mg three times a day at three meals 1 week later. The dose of metformin was 500 mg three times a day throughout the study. The patients have already made adjustment to their lifestyle before the enrollment and did not change their previous lifestyle during the 12-week hypoglycemic intervention. Finally, 54 subjects from MET group and 61 from ACA group completed the whole study.

The study was approved by the Ethics Committee of Shanghai Jiao Tong University Affiliated Sixth People’s Hospital and was in accordance with the 1964 Declaration of Helsinki. Informed consent was obtained from each subject at the beginning of the study.

### Anthropometric and biochemical measurements

Each subject had standardized meal tests at baseline and at the 12-week follow-up visit. Fasting blood samples were collected after an 8-hour overnight fast and postprandial blood was collected 2-hour later. The standard meal test was standardized instant noodles containing 69.3 g of carbohydrates, 9.3 g of protein, and 1.5 g of fat [15]. Blood pressure, height and waist were measured. BMI = weight (kg)/height(m)^2^.

Plasma glucose including fasting plasma glucose (FPG) and 2-h postprandial plasma glucose (2-h PG) levels were measured by glucose oxidase method. HbA1c was measured by using high-performance liquid chromatography with a VARIANT II Hemoglobin A1c analyzer (Bio-Rad Laboratories, Hercules, CA). Triglycerides (TG), total cholesterol (TC), high-density lipoprotein cholesterol (HDL-c), and low-density lipoprotein cholesterol (LDL-c) were ﻿determined by applying standard enzymatic methods using a biochemical analyzer (7600–120; Hitachi, Tokyo, Japan). The criteria for the serum sample included: 1) the volume of the serum sample was enough for the assay; 2) serum sample was stored properly. For fasting serum GDF15 levels, 51 of subjects from MET group and 53 subjects from ACA group were measured at the baseline and endpoint by quantitative sandwich enzyme-linked immunosorbent assay (R&D Systems, Minneapolis, USA).

### Statistical analyses

Statistical analyses were performed in SPSS version 24.0 (SPSS, Inc., Chicago, IL). The Kolmogorov-Smirnov test was performed to determine normality of the data distribution. Variables with a normal distribution were presented as means ± standard deviation (SD), and variables with a skewed distribution were presented as median (interquartile range). Differences of the parameters before and after treatment were compared by the paired *t*-test or Wilcoxon signed rank test. Differences between groups were compared using the student *t*-test or the Mann-Whitney test. The correlation between clinical parameters and the change of serum GDF15 levels were assessed by Spearman correlation coefficient and analysis of covariance (ANCOVA). A *P* value of < 0.05 (two-tailed) was considered statistically significant.

## Results

### Basic characteristics of the subjects

We finally enrolled a total of 51 subjects from MET group and 53 subjects from ACA group in the present analysis. The characteristics of the participants at baseline and at the endpoint are presented in Table 1. Compared with baseline levels, glycemic control parameters (FPG, 2-h PG, HbA1c) all significantly improved in both MET and ACA group after a 12-week therapy (all *P* < 0.01, Table 1). Body weight, BMI and waist circumference did not change in MET group (all *P* > 0.05, Table 1).

**Table 1:** Clinical characteristics of the participants at the baseline and endpoint

	Metformin (***N*** = 51)			Acarbose (***N*** = 53)		
	Baseline	Endpoint	*P*	Baseline	Endpoint	*P*
Sex (male/female)	28/23	–	–	32/21	–	–
Age (year)	60.00 (53.00, 64.00)	–	–	63.00 (57.00, 67.00)	–	–
Diabetes duration (year)	14.00 (10.00, 17.00)	–	–	16.00 (11.00, 20.00)	–	–
Body weight (kg)	74.13 ± 10.22	73.65 ± 10.14	0.181	69.66 ± 9.23	68.63 ± 9.34	< 0.001
BMI (kg/m^2^)	26.37 ± 2.80	26.21 ± 2.83	0.197	25.31 ± 2.42	24.94 ± 2.43	< 0.001
Waist circumference (cm)	91.11 ± 10.66	92.35 ± 9.61	0.186	88.68 ± 8.33	88.49 ± 8.10	0.736
SBP (mmHg)	137.00 ± 13.00	133.00 ± 16.00	0.107	136.00 ± 16.00	130.00 ± 14.00	0.005
DBP (mmHg)	81.00 (76.00, 86.00)	80.00 (76.00, 85.00)	0.587	81.00 (73.00, 85.00)	77.00 (71.00, 82.00)	0.089
TC (mmol/L)	4.97 ± 1.18	4.82 ± 1.06	0.386	4.93 ± 0.90	4.97 ± 1.08	0.781
TG (mmol/L)	1.63 (1.10, 2.25)	1.55 (1.02, 2.18)	0.636	1.33 (0.95, 2.10)	1.19 (0.89, 1.53)	0.025
HDL-c (mmol/L)	1.09 (0.96, 1.35)	1.14 (0.95, 1.30)	0.881	1.17 (1.03, 1.46)	1.15 (0.97, 1.33)	0.089
LDL-c (mmol/L)	2.89 ± 0.99	2.76 ± 0.83	0.345	2.94 ± 0.76	3.00 ± 0.85	0.552
FPG (mmol/L)	9.91 (8.79, 12.16)	8.28 (7.29, 9.76)	< 0.001	10.29 (8.88, 12.14)	8.71 (7.49, 10.42)	0.003
2-h PG (mmol/L)	20.03 ± 3.31	15.48 ± 3.53	< 0.001	20.37 ± 3.72	11.60 ± 3.75	< 0.001
HbA1c (%)	8.40 (7.70, 9.00)	7.50 (7.00, 8.10)	< 0.001	8.50 (7.80, 9.10)	7.50 (7.00, 8.10)	< 0.001

Data are expressed as mean ± SD or median (interquartile range)

*Abbreviations*: *BMI* Body mass index, *SBP* Systolic blood pressure, *DBP* Diastolic blood pressure, *TC* Total cholesterol, *TG* Triglycerides, *HDL-c* High-density lipoprotein cholesterol, *LDL-c* Low-density lipoprotein cholesterol, *FPG* Fasting plasma glucose, *2-h PG* 2-h postprandial plasma glucose, *HbA1c* Glycated hemoglobin A1c

### Change of serum GDF15 levels before and after treatment

**Fig. 1:**
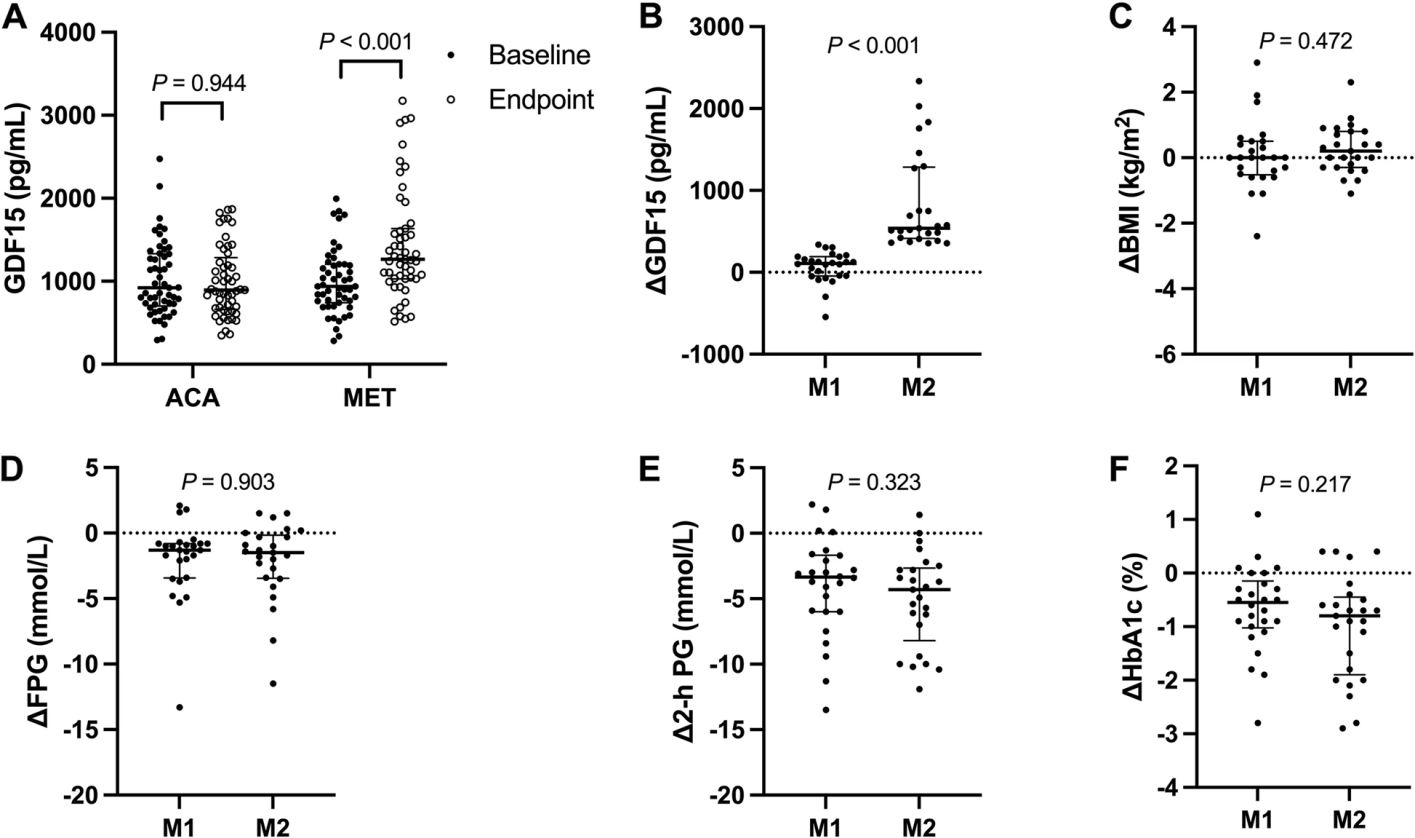
**A** The comparison of serum GDF15 levels between baseline and endpoint of ACA group and MET group; **B**-**F** The comparison of ΔGDF15, ΔBMI, ΔFPG, Δ2-h PG and ΔHbA1c levels between M1 and M2 subgroup (divided by the median of ΔGDF15 in MET group; M1, ΔGDF15 ≤ 338.50 pg/mL; M2, ΔGDF15 > 338.50 pg/mL). GDF15, growth differentiation factor 15; BMI, body mass index; FPG, fasting plasma glucose; 2-h PG, 2-h postprandial plasma glucose; HbA1c, glycated hemoglobin A1c

After a 12-week treatment, serum GDF15 levels significantly increased in MET group [baseline vs. endpoint, 936.70 (741.00, 1205.40) pg/mL vs. 1265.20 (1027.90, 1634.00) pg/mL, *P* < 0.001, Fig. 1A], but not in ACA group [baseline vs. endpoint, 920.60 (701.45, 1332.55) pg/mL vs. 893.80 (663.25, 1284.05) pg/mL, *P* = 0.944, Fig. 1A]. There were no differences of serum GDF15 levels between the MET group and ACA group at baseline (*P* = 0.946), while serum GDF15 levels at the endpoint were much higher in MET group than those in ACA group (*P* < 0.001).

### Relationship between serum GDF15 levels and glycemic parameters in the MET group

Since the differences of serum GDF15 levels after treatment were only discovered in the MET group, we divided the MET group into two subgroups (M1 group, *n* = 26; M2 group, *n* = 25) according to the median of ΔGDF15 levels [338.50 (101.10, 538.00) pg/mL], to further explore the relationship between ΔGDF15 and glycemic control parameters. The clinical characteristics of the participants of M1 and M2 subgroups are presented in Table 2. Body weight, BMI and waist circumference showed no significant differences in M1 and M2 group (all *P* > 0.05, Table 2). TG levels were lower while HDL-c levels were higher in M2 group at baseline (all *P* < 0.05, Table 2). There were no significant differences between M1 and M2 subgroup in glycemic control parameters (FPG, 2-h PG and HbA1c) at both baseline and endpoint (all *P* > 0.05, Table 2).

**Table 2:** Clinical characteristics of the participants of M1 and M2 subgroups

	M1 (***N*** = 26)			M2 (***N*** = 25)		
	Baseline	Endpoint	*P*	Baseline	Endpoint	*P*
Sex (male/female)	16/10	–	–	12/13	–	–
Age (year)	60.00 (55.00, 62.00)	–	–	62.00 (49.00, 67.00)	–	–
Diabetes duration (year)	14.00 (10.00, 17.00)	–	–	13.00 (10.00, 16.00)	–	–
Body weight (kg)	75.34 ± 10.15	75.10 ± 10.20	0.685	72.88 ± 10.35	72.14 ± 10.06	0.072
BMI (kg/m^2^)	26.63 ± 3.10	26.55 ± 3.24	0.716	26.11 ± 2.50	25.85 ± 2.35	0.094
Waist circumference (cm)	91.44 ± 9.55	92.46 ± 7.65	0.473	90.76 ± 11.90	92.24 ± 11.46	0.243
SBP (mmHg)	137.00 ± 12.00	131.00 ± 15.00	0.092	138.00 ± 15.00	136.00 ± 16.00	0.608
DBP (mmHg)	80.00 ± 6.00	80.00 ± 7.00	0.980	82.00 ± 7.00	81.00 ± 8.00	0.305
TC (mmol/L)	4.80 ± 1.00	4.68 ± 1.10	0.587	5.14 ± 1.33	4.97 ± 1.02	0.510
TG (mmol/L)	1.90 (1.28, 3.03)	1.58 (1.07, 2.50)	0.041	1.26 (0.79, 1.82) ^*^	1.55 (1.01, 1.94)	0.087
HDL-c (mmol/L)	1.04 (0.92, 1.21)	1.13 (0.89, 1.25)	0.096	1.14 (1.00, 1.54) ^*^	1.22 (0.99, 1.35)	0.277
LDL-c (mmol/L)	2.62 ± 0.78	2.69 ± 0.93	0.683	3.17 ± 1.12	2.83 ± 0.72	0.121
FPG (mmol/L)	9.91 (9.08, 12.22)	8.18 (6.82, 10.36)	0.001	10.25 (8.49, 11.96)	8.28 (7.46, 9.61)	0.001
2-h PG (mmol/L)	18.74 (17.55, 21.06)	14.52 (12.67, 18.18)	< 0.001	20.36 (18.06, 22.72)	16.09 (14.64, 17.57)	< 0.001
HbA1c (%)	8.20 ± 0.70	7.50 ± 0.60	< 0.001	8.60 ± 0.90	7.60 ± 0.80	< 0.001
GDF15 (pg/mL)	983.05 (694.70, 1324.85)	1040.65 (853.75, 1333.93)	0.016	897.40 (769.90, 1171.30)	1559.70 (1247.35, 2416.00) ^**^	< 0.001

Data are expressed as mean ± SD or median (interquartile range)

M1 subgroup, ΔGDF15 ≤ 338.50 pg/mL; M2 subgroup, ΔGDF15 > 338.50 pg/mL

Compared to M1 group: ^*^, *P* < 0.05; ^**^, *P* < 0.01

*Abbreviations*: *BMI* Body mass index, *SBP* Systolic blood pressure, *DBP* Diastolic blood pressure; *TC* Total cholesterol, *TG* Triglycerides, *HDL-c* High-density lipoprotein cholesterol, *LDL-c* Low-density lipoprotein cholesterol; *FPG* Fasting plasma glucose, *2-h PG* 2-h postprandial plasma glucose, *HbA1c* Glycated hemoglobin A1c, *GDF15* Growth differentiation factor 15

Serum GDF15 levels at baseline were 983.05 (694.70, 1324.85) and 897.40 (769.90, 1171.30) in M1 and M2 group (*P* = 0.692), respectively. Serum GDF15 levels at endpoint were 1040.65 (853.75, 1333.93) and 1559.70 (1247.35, 2416.00) in M1 and M2 group (*P* < 0.001), respectively. The median (interquartile range) of ΔGDF15 of M1 and M2 subgroup were 108.15 (− 44.23, 191.68) and 538.00 (415.00, 1283.55), respectively (Fig. 1B, *P* < 0.001). There were no significant differences of ΔBMI, ΔFPG, Δ2-h PG and ΔHbA1c between the M1 and M2 subgroup [ΔBMI, 0.00 (− 0.49, 0.50) vs. 0.17 (− 0.31, 0.76); ΔFPG, − 1.3 (− 3.40, − 0.76) vs. -1.49 (− 3.47, − 0.13); Δ2-h PG, − 3.32 (− 6.02, − 1.68) vs. -4.26 (− 8.21, − 2.63); ΔHbA1c, − 0.55 (− 1.03, − 0.15) vs. -0.80 (− 1.90, − 0.45), all *P* > 0.05, Fig. 1C-F).

Spearman correlation coefficient was used to analyze the possible relationship between glycemic control parameters and results showed that ΔGDF15 was not correlated to ΔFPG, Δ2-h PG and ΔHbA1c (all *P* > 0.05, Table 3). To further verify the relationship, analysis of covariance after adjustment for baseline HbA1c levels showed that ΔGDF15 was also not correlated to ΔFPG, Δ2-h PG and ΔHbA1c (ΔFPG, *P* = 0.682; Δ2-h PG, *P* = 0.704; ΔHbA1c, *P* = 0.725).

**Table 3:** Spearman correlation analysis of ΔGDF15 and the change of clinical parameters in MET group

	ΔGDF15	
	*r*	*P*
ΔFPG (mmol/L)	0.075	0.602
Δ2-h PG (mmol/L)	−0.131	0.361
ΔHbA1c (%)	−0.166	0.245

*Abbreviations*: *GDF15* Growth differentiation factor 15, *FPG* Fasting plasma glucose, *2-h PG* 2-h postprandial plasma glucose, *HbA1c* Glycated hemoglobin A1c

## Discussion

It has been reported that GDF15 was a promising biomarker for metformin treatment and could reflect the dosage of metformin treatment at the same time [10, 16]. In a nested case-control study, Natali et al. [17] found that in patients with diabetes mellitus, metformin treatment was associated with a 40% rise in serum GDF15 levels. Although these cross-sectional studies have focused on GDF15 and metformin, our study first investigated the change of serum GDF15 levels before and after metformin treatment in patients with type 2 diabetes mellitus. We found that compared with baseline levels, serum GDF15 levels increased about 35% after a 12-week metformin treatment in patients with type 2 diabetes mellitus. Moreover, our results showed that after acarbose treatment, serum GDF15 levels did not change. It is similar with previous studies that serum GDF15 levels were not associated with other hypoglycemia therapy [10, 17].

Although the increase of GDF15 levels was a unique characteristic of metformin treatment, the change of GDF15 in previous studies was discovered to be mainly associated with weight loss so far [18]. A previous study focusing on people without diabetes mellitus revealed that metformin treatment was associated with significantly increased levels of circulation GDF15 with lost about 3.5% of body weight [12]. There still remains a paucity of information on the relationship between GDF15 and the glucose-lowering effect of metformin in patients with type 2 diabetes mellitus. The present study first found that the increase of serum GDF15 levels after metformin intervention was not related to the improvement of glycemic control parameters (ΔFPG, Δ2-h PG or ΔHbA1c) in patients with type 2 diabetes mellitus. Our results also showed that serum GDF15 levels did not increase in nearly half of the subjects after a 12-week metformin treatment, while serum GDF15 levels of the other half increased approximately 70%. However, the changes of glycemic control parameters were similar in the two subgroups (divided according to the median of ΔGDF15), which further proved that the elevation of serum GDF15 levels was not directly associated with glucose metabolism improvement in patients with type 2 diabetes mellitus. Still, reasons for the differences of ΔGDF15 between these two subgroups after metformin intervention need further detailed investigation.

To be noted, body weight of the MET group did not change after the intervention. Possible reasons may be the features of participants in our study. Firstly, the participants included were mostly overweight, not obese. Moreover, the improvement of the glycemic control in MET group may have prevented the loss of nutrition and partly neutralized the weight-loss effect of metformin. Without weight changes, this study population was definitely a proper model to explore the underlying relationship between GDF15 and the glucose-lowering effect of metformin. Previous animal studies showed that overexpression of GDF15 levels in mice led to decreased food intake, body weight and improved glucose metabolism [19, 20]. Taking the results from our study into consideration, the improved glucose metabolism in animal studies may be secondary to the decreased food intake and body weight.

There are some limitations of the present study. First, this was a pilot study, so the sample size was relatively small. In addition, we did not evaluate GDF15 levels after the standard meal. Therefore, further large-scale studies with more intense investigation on the change of serum GDF15 levels are needed to precisely elucidate the relationship among GDF15, glycemic control and metformin treatment.

In conclusion, the increase of serum GDF15 levels was an indicator of metformin treatment. However, the increase was not associated with the improvement of glycemic control in patients with type 2 diabetes mellitus.

## Data Availability

The datasets generated during and/or analyzed during the current study are not publicly available but are available from the corresponding author on reasonable request.

## Abbreviations

GDF15: Growth differentiation factor 15
BMI: Body mass index
HbA1c: Glycated hemoglobin A1c
FPG: Fasting plasma glucose
2-h PG: 2-h postprandial plasma glucose
SBP: Systolic blood pressure
DBP: Diastolic blood pressure
TC: Total cholesterol
TG: Triglycerides
HDL-c: High-density lipoprotein cholesterol
LDL-c: Low-density lipoprotein cholesterol
